# Incidence and risk factors of surgical site infection following colorectal surgery in China: a national cross-sectional study

**DOI:** 10.1186/s12879-020-05567-6

**Published:** 2020-11-12

**Authors:** Xufei Zhang, Zhiwei Wang, Jun Chen, Peige Wang, Suming Luo, Xinjian Xu, Wei Mai, Guangyi Li, Gefei Wang, Xiuwen Wu, Jianan Ren

**Affiliations:** 1 Research Institute of General Surgery, Jinling Hospital, Nanjing Medical University, Nanjing, 210002 People’s Republic of China; 2 Department of Surgery, The Second Affiliated Hospital, School of Medicine, Zhejiang University, Hangzhou, 310009 People’s Republic of China; 3 Research Institute of General Surgery, Jinling Hospital, Medical School of Nanjing University, Nanjing, 210002 People’s Republic of China; 4 Department of Emergency Surgery, The Affiliated Hospital of Qingdao University, Qingdao, 266000 People’s Republic of China; 5 Department of Emergency Trauma Surgery, The People’s Hospital of Xinjiang Uygur Autonomous Region, Urumqi, 830001 People’s Republic of China; 6 Department of General Surgery, The First Affiliated Hospital of Xinjiang Medical University, Urumqi, 830054 People’s Republic of China; 7 Department of Gastrointestinal Surgery, The People’s Hospital of Guangxi Zhuang Autonomous Region, Nanning, 530021 People’s Republic of China; 8 Department of Gastrointestinal Surgery, The People’s Hospital of Hunan, Changsha, 410005 People’s Republic of China

**Keywords:** SSI, Colorectal surgery, Prevalence, Risk factors, Multicenter study

## Abstract

**Purposes:**

Surgical site infection (SSI) after colorectal surgery is a frequent complication associated with the increase in morbidity, medical expenses, and mortality. To date, there is no nationwide large-scale database of SSI after colorectal surgery in China. The aim of this study was to determine the incidence of SSI after colorectal surgery in China and to further evaluate the related risk factors.

**Methods:**

Two multicenter, prospective, cross-sectional studies covering 55 hospitals in China and enrolling adult patients undergoing colorectal surgery were conducted from May 1 to June 30 of 2018 and the same time of 2019. The demographic and perioperative characteristics were collected, and the main outcome was SSI within postoperative 30 days. Multivariable logistic regressions were conducted to predict risk factors of SSI after colorectal surgery.

**Results:**

In total, 1046 patients were enrolled and SSI occurred in 74 patients (7.1%). In the multivariate analysis with adjustments, significant factors associated with SSI were the prior diagnosis of hypertension (OR, 1.903; 95% confidence interval [CI], 1.088–3.327, *P* = 0.025), national nosocomial infection surveillance risk index score of 2 or 3 (OR, 3.840; 95% CI, 1.926–7.658, *P* < 0.001), laparoscopic or robotic surgery (OR, 0.363; 95% CI, 0.200–0.659, *P* < 0.001), and adhesive incise drapes (OR, 0.400; 95% CI, 0.187–0.855, *P* = 0.018). In addition, SSI group had remarkably increased length of postoperative stays (median, 15.0 d versus 9.0d, *P* < 0.001), medical expenses (median, 74,620 yuan versus 57,827 yuan, *P* < 0.001), and the mortality (4.1% versus 0.3%, *P* = 0.006), compared with those of non-SSI group.

**Conclusion:**

This study provides the newest data of SSI after colorectal surgery in China and finds some predictors of SSI. The data presented in our study can be a tool to develop optimal preventive measures and improve surgical quality in China.

**Supplementary Information:**

The online version contains supplementary material available at 10.1186/s12879-020-05567-6.

## Introduction

To date, surgical site infection (SSI) is still one of the most common types of health care-related infection, especially in middle-income and low-income countries [1]. Moreover, SSI is accompanied with the prolongation of hospital stays and the increase of related medical expenses [2]. In addition, the occurrence of SSI continues to be an important role in nosocomial morbidity and mortality [3]. Thus, the prevention of SSI remains a pressing concern.

Because of the existence of numerous microbes in the rectum and colon, the SSI incidence of colorectal surgery is usually higher than those observed in other types of surgeries [4, 5]. Little multicenter surveillance focusing on SSI after colorectal surgery has been carried out in China. In 2019, Liu et al. [6] reported SSI and its risk factor in radical resection of colon or rectal carcinoma, but patients were rolled from January 2015 to June 2016. At present, there is a lack of recent studies to reflect the current situation of SSI after colorectal surgery in China in recent years.

Our group has conducted two multicenter, prospective, cross-sectional studies enrolling adult patients (> 18 years) who received colorectal surgery from May 1 to June 30 of 2018 and the same time of 2019 in China. This nationwide data aims to determine the incidence rate and related risk factors of SSI after colorectal surgery.

## Patients and methods

### Study design

Two multicenter, prospective, cross-sectional studies were conducted from May 1 to June 30 of 2018 and the same time of 2019, and enrolled adult patients (> 18 years) who received colorectal surgery in the department of general surgery of 55 hospitals in China during these periods (Supplementary file 1). The follow-up period was defined as 30 days after surgery. The exclusion criteria were pregnancy, patients undergoing urological, transplantation or gynecological surgery. The ethics committees of all institutions involved in the study approved our study. All enrolled patients should provide written informed consent ahead of their participation in the study. For data collecting, we only included those patients who have agreed to use the information from their medical records for the purpose of scientific research.

### Data collection

Aside from baseline variables, we collected other data which may be related to the likelihood of SSI occurrence. Firstly, each hospital identified patients who met the inclusion criteria and could comply with follow-up, and collected their essential data. Secondly, we had specialized team members to evaluate the whole data collection process and the accuracy of all data.

Baseline variables of enrolled patients covered gender, age, body mass index (BMI), American Society of Anesthesiologists (ASA) physical status score, diagnosis, the prior diagnosis of diabetes mellitus, hypertension, chronic renal dysfunction (renal failure or dialysis), chronic hepatic dysfunction (abnormal concentrations of liver enzymes, hepatocellular carcinoma, cirrhosis or hepatitis), chronic cardiac dysfunction (heart failure, myocardial infarction, or previous cardiac surgery), tuberculosis, use of immunosuppressive medication, smoking status (nonsmoker, former smoker, or current smoker), preoperative blood biochemical parameters (albumin, hemoglobin and fasting blood glucose; collected at the preoperative day of surgery), and length of preoperative stay.

Patient perioperative characteristics covered type of surgery based on the urgency of surgery (emergency surgery or elective surgery), surgical site (colon or rectum), timing of hair removal (none, night before surgery, or day of surgery), surgical wound classes (dirty, clean-contaminated, or contaminated), ways of bowel preparation (oral antibiotics bowel preparation [OABP] without mechanical bowel preparation [MBP], MBP without OABP, or OABP combined with MBP), type of surgical hand preparation (scrubbing or disinfectant), surgery approach (open surgery, laparoscopy surgery or robotic surgery), incisional protection (adhesive incise drapes, wound edge protector, or gauze which is put around the incision to avoid the friction of surgical instruments on the incision skin and subcutaneous tissue), type of fluids for incisional wound irrigation (povidone-iodine solution, hydrogen peroxide solution, saline, or something else), fascial or muscle suture materials (silk suture, absorbable sutures, or antimicrobial-coated and absorbable sutures), skin closure materials (silk suture, absorbable sutures, skin staples, or something else), duration of surgery, grade of lead surgeon on the basis of their professional titles, colostomy/ileostomy, and the national nosocomial infections surveillance (NNIS) risk index.

The NNIS risk index is an internationally recognized method for stratifying surgical risk, while the NNIS risk index [7] varied from 0 to 3 by assessing three variables: duration of surgery, surgical wound class and ASA score. Each variable’s cutoff values were a contaminated or dirty surgical incision, the operative duration of 225 min and an ASA score of 3, with 1 point evaluated if any one variable was over the cutoff value.

In this study, the primary outcome was the occurrence of SSI within postoperative 30 days defined by Center for Disease Control criteria [8], including organ-space infections, deep or superficial incisional infections. The follow-up was carried out via standard telephone interview or review of the readmission records if patients were discharged from hospital after less than postoperative 30 d. If patients had more than one type of SSI within 30 days, only a single form of SSI with the deepest anatomy was included in subsequent analysis. In addition, once SSI happened, the bacterial culture of secretion, the pus, pelvic puncture fluid, or distal catheter would be conducted, which depended on the criteria of each hospital.

The secondary outcomes were duration of total hospital stay and postoperative stay, costs, and postoperative 30-day mortality.

### Statistical analysis

Results were shown as the mean ± standard deviation (SD) or median with interquartile range (IQR), as appropriate. Comparisons of all continuous variables between the two groups were conducted by using the Mann-Whitney U-test or Student’s t-test depending on Gaussian distribution. Comparisons of categorical variables were conducted using Fisher’s exact test or chi-square test, as appropriate. The criterion of statistical significance was *P* < 0.05.

Variables with statistical significance in univariable logistic regressions would be included into multivariable logistic regression analysis to identify the independent risk factors of SSI within postoperative 30 days.

We used SPSS v24 software to analyze all data.

## Results

In total, 1046 patients receiving colorectal surgery were enrolled in this study, of which 74 patients developed SSI within postoperative 30 days, namely the incidence rate of SSI for 7.1%. For patients with SSI, 24 developed organ-space infections, 18 developed deep incisional infections, and 32 developed superficial incisional infections. The demographics of the enrolled patients are exhibited in Table 1. There was no difference between non-SSI group and SSI group on gender, age, BMI, length of preoperative stay or smoking history. The most common comorbidities were hypertension and diabetes mellitus, and the two groups significantly differed in the presence or absence of hypertension (*P* = 0.006). Moreover, the incidence of SSI in patients with higher preoperative blood glucose was significantly increased (*P* = 0.025). In addition, the overall rate of low albumin concentration (< 3.5 g/dl) in non-SSI group and SSI group was 20.5 and 43.2% (*P* < 0.001).

**Table 1:** Demographics of included patients

Variables	Total	Non-SSI group	SSI group	***p*** value
N (%)	1046 (100%)	972 (92.9%)	74 (7.1%)	
Age, years, median (IQR)	59.0 (52.0–70.0)	62.0 (53.0–70.0)	60.5 (50.0–71.0)	0.841
Gender (%)				0.302
Male	619 (59.2%)	571 (58.7%)	48 (64.9%)	
Female	427 (40.8%)	401 (41.3%)	26 (35.1%)	
BMI, median (IQR)	23.0 (20.3–25.4)	23.0 (20.3–25.2)	23.9 (19.9–26.3)	0.421
Comorbidity (%)				
Diabetes mellitus	128 (12.2%)	115 (11.8%)	13 (17.6%)	0.147
Hypertension	257 (24.6%)	229 (23.6%)	28 (37.8%)	**0.006**
Chronic liver disease	20 (1.9%)	20 (2.1%)	0 (0%)	0.390
Chronic kidney disease	9(0.9%)	9 (0.9%)	0 (0%)	1.00
Chronic heart disease	40 (3.8%)	34 (3.5%)	6 (8.1%)	0.057
Tuberculosis	5 (0.5%)	3 (0.3%)	2 (2.7%)	**0.043**
Steroid use	9 (0.9%)	8 (0.8%)	1 (1.4%)	0.485
Immunosuppressive medication	11 (1.1%)	9 (0.9%)	2 (2.7%)	0.180
Smoking history (%)				0.980
No	839 (80.2%)	780 (80.2%)	59 (79.7%)	
Former	45 (4.3%)	42 (4.3%)	3 (4.1%)	
Current	162 (15.5%)	150 (15.4%)	12 (16.2%)	
Hemoglobin, g/dl (%)				0.183
≥ 11	762 (72.8%)	713 (73.4%)	49 (66.2%)	
< 11	284 (27.2%)	259 (26.6%)	25 (33.8%)	
Albumin, g/dl (%)				**< 0.001**
≥ 3.5	815 (77.9%)	773 (79.5%)	42 (56.8%)	
< 3.5	231 (22.1%)	199 (20.5%)	32 (43.2%)	
Blood glucose, mg/dl (%)				**0.025**
< 80	181 (17.3%)	168 (17.3%)	13 (17.6%)	
80 ~ 150	771 (73.7%)	723 (74.4%)	48 (64.9%)	
> 150	94 (9.0%)	81 (8.3%)	13 (17.6%)	
LPS, day, median (IQR)	6 (3.5–9.0)	6 (3–9)	7.5 (4–9)	0.130

*IQR* interquartile range, *BMI* body mass index, *LPS* Length of preoperative stay

The perioperative information of the patients is summarized in Table 2. Of note, the SSI incidence varied significantly based on surgical sites, of which colon surgery had higher SSI incidence (9.0% versus 4.0%). The emergency surgery accounted for 6.5% of all enrolled patients. The clean-contaminated wound was the most common type of wound classes, and patients with an ASA score of 1 or 2 were over 70%. The incidence of SSI in patients with a clean-contaminated wound was significantly lower than those with a contaminated or dirty wound (6.0% versus 25.9%, *P* **<** 0.001). Regardless the timing of hair removal (the day of surgery or the night before surgery), patients undergoing hair removed had lower SSI incidence compared with those without hair removal (*P* = 0.028). In addition, the most common method of bowel preparation was MBP alone, and the incidence of SSI in patients with MBP alone was remarkably lower compared with those undergoing no bowel preparation (*P* **<** 0.001).

**Table 2:** Perioperative characteristics of included patients

Variables	Total (***n*** = 1046)	Non-SSI group (***n*** = 972)	SSI group (***n*** = 74)	***p*** value
Type of surgery (%)				**0.002**
Colon surgery	641 (61.3%)	583 (60.0%)	58 (78.4%)	
Rectal surgery	405 (38.7%)	389 (40.0%)	16 (21.6%)	
Urgency of surgery (%)				**< 0.001**
Elective	978 (93.5%)	922 (94.9%)	56 (75.7%)	
Emergency	68 (6.5%)	50 (5.1%)	18 (24.3%)	
ASA score (%)				**0.001**
1 or 2	818 (78.2%)	772 (79.4%)	46 (62.2%)	
3 or 4	228 (21.8%)	200 (20.6%)	28 (37.8%)	
Surgical wound class (%)				**< 0.001**
Clean-contaminated	992 (94.9%)	932 (95.9%)	60 (81.1%)	
Contaminated or dirty	54 (5.2%)	40 (4.1%)	14 (18.9%)	
Timing of hair removal (%)				**0.028**
None	188 (18.0%)	167 (17.2%)	21 (28.4%)	
Night before surgery	672 (64.2%)	634 (65.2%)	38 (51.4%)	
Day of surgery	186 (17.8%)	171 (17.6%)	15 (20.3%)	
Hair removal				**0.016**
No	188 (18.0%)	167 (17.2%)	21 (28.4%)	
Yes	858 (82.0%)	805 (82.8%)	53 (71.6%)	
Bowel preparation (%)				**< 0.001**
None	331 (31.6%)	294 (30.2%)	37 (50.0%)	
MBP only	575 (55.0%)	549 (56.5%)	26 (35.1%)	
OABP only	42 (4.0%)	36 (3.7%)	6 (8.1%)	
MBP + OABP	98 (9.4%)	93 (9.6%)	5 (6.8%)	
Hand preparation (%)				0.209
Disinfectant	831 (79.4%)	768 (79.0%)	63 (85.1%)	
Scrubbing	215 (20.6%)	204 (21.0%)	11 (14.9%)	
Approach (%)				**< 0.001**
Open	391 (37.4%)	341 (35.1%)	50 (67.6%)	
Laparoscopic or robotic	655 (62.6%)	631 (64.9%)	24 (32.4%)	
Incisional protection (%)				0.151
None	211 (20.2%)	191 (19.7%)	20 (27.0%)	
Gauze	121 (11.6%)	113 (11.6%)	8 (10.8%)	
Adhesive incise drapes	340 (32.5%)	324 (33.3%)	16 (21.6%)	
Wound edge protector	374 (35.8%)	344 (35.4%)	30 (40.5%)	
Skin closure materials (%)				**0.027**
Silk sutures	627 (60.0%)	579(59.6%)	48(64.9%)	
Absorbable sutures	264 (25.2%)	253(26.0%)	11(14.9%)	
Skin staples	120 (11.5%)	111(11.4%)	9(12.2%)	
other	35 (3.3%)	29(3.0%)	6(8.1%)	
Fascial or muscle suture materials (%)				0.545
Silk sutures	153 (14.6%)	140 (14.4%)	13 (17.6%)	
Absorbable sutures	408 (39.0%)	377 (38.8%)	31 (41.9%)	
antimicrobial-coated and absorbable sutures	485 (46.4%)	455 (46.8%)	30 (40.5%)	
Incisional wound irrigation (%)				**0.037**
None	81 (7.7%)	72 (7.4%)	9 (12.2%)	
Povidone-iodine solution	182 (17.4%)	172 (17.7%)	10 (13.5%)	
Hydrogen peroxide solution	51 (4.9%)	48 (4.9%)	3 (4.1%)	
Saline	701 (67.0%)	655 (67.4%)	46 (62.2%)	
Other	31 (3.0%)	25 (2.6%)	6 (8.1%)	
Colostomy/ileostomy				**< 0.001**
No	920 (88.0%)	869 (89.4%)	51 (68.9%)	
Yes	126 (12.0%)	103 (10.6%)	23 (31.1%)	
Grade of lead surgeon (%)				0.068
Senior	694 (66.3%)	651 (67.0%)	43 (58.1%)	
Middle	304 (29.1%)	280 (28.8%)	24 (32.4%)	
Junior	48 (4.6%)	41 (4.2%)	7 (9.5%)	
Surgical duration, min, median (IQR)	180.0 (130.0–225.0)	180.0 (130.0–220.0)	193.5 (143.8–254.3)	**0.019**
NNIS risk index				**< 0.001**
0 or 1	969 (92.6%)	916 (94.2%)	53 (71.6%)	
2 or 3	77 (7.4%)	56 (5.8%)	21 (28.4%)	

*ASA* American society of anesthesiologists physical status classification system, *MBP* mechanical bowel preparation, *OABP* oral antibiotic bowel preparation, *NNIS* national nosocomial infections surveillance

Regarding the approach of surgery, laparoscopic or robotic surgery had lower SSI incidence than open surgery (3.7% versus 12.8%, *P* **<** 0.001). Silk sutures were the most commonly used skin closure materials, but absorbable sutures had the lowest SSI incidence in comparison with other sutures (*P* = 0.027). There was no difference among patients receiving different fascial or muscle suture materials when comparing SSI. Among materials of incisional wound irrigation, saline was the most commonly used material. In patients with colostomy/ileostomy, 18.3% developed SSI (*P* **<** 0.001). In addition, patients with the higher NNIS risk index scores had the remarkably higher incidence rate of SSI (*P* **<** 0.001).

The secondary outcomes of studies have been exhibited in Table 3. The median time of SSI occurrence is 5.5d (IQR: 3.0–9.0 d) after surgery. The SSI group had the remarkably longer lengths of total hospital stays compared with those in the non-SSI group (22.5 d versus 15.0 d, *P* < 0.001). Moreover, compared with the non-SSI group, there was the longer length of postoperative stays in the SSI group (15.0 d versus 9.0 d, *P* < 0.001). The medical expenses of the non-SSI group were also lower compared with those of the SSI group (*P* **<** 0.001). During the study, a total of six patients died, and the mortality in the SSI group was also remarkably higher compared with that in the non-SSI group (*P* = 0.006).

**Table 3:** Outcomes of included patients

Variables	Total (***n*** = 1046)	Non-SSI group (***n*** = 972)	SSI group (***n*** = 74)	***p*** value
Length of hospital stay, day, median (IQR)	16.0 (12.0–21.0)	15.0 (12.0–21.0)	22.5 (17.0–33.0)	**< 0.001**
Length of postoperative stay, day, median (IQR)	9.0 (7.0–12.0)	9.0 (7.0–12.0)	15.0 (11.0–24.0)	**< 0.001**
30-day mortality, n, (%)	6(0.6%)	3 (0.3%)	3 (4.1%)	**0.006**
Medical cost, yuan, median (IQR)	58,982 (42972–79,262)	57,827 (42000–77,919)	74,620 (59078–107,612)	**< 0.001**
Surgery to SSI time, day, median (IQR)	–	–	5.5 (3.0–9.0)	–
SSI type, n, (%)	–	–		–
Superficial incisional infection	–	–	32 (43.2%)	–
Deep incisional infection	–	–	18 (24.3%)	–
Organ-space infection	–	–	24 (32.4%)	–
Pathogen, n, (%)				
*Escherichia coli*	–	–	25(33.8%)	–
*Klebsiella pneumoniae*	–	–	4(5.4%)	–
*Enterococcus faecalis*	–	–	3(4.1%)	–
*Enterococcus faecium*	–	–	2(2.7%)	–
*Morganella morganii*	–	–	2(2.7%)	–
*Candida albicans*	–	–	2(2.7%)	–
*Pseudomonas aeruginosa*	–	–	1(1.4%)	–
*Acinetobacter baumanii*	–	–	1(1.4%)	–
*Staphylococcus aureus*	–	–	1(1.4%)	–
*Proteus mirabilis*	–	–	1(1.4%)	–
*Enterobacter cloacae*	–	–	1(1.4%)	–
*Staphylococcus epidermidis*	–	–	1(1.4%)	–
Negative	–	–	21(28.4%)	–
None of germiculture	–	–	17(23.0%)	–

Table 4 shows the logistic regression analysis. The risk factors significantly associated with the occurrence of SSI were hypertension, tuberculosis, low albumin (< 3.5 g/dl), blood glucose > 150 mg/dl, colon surgery, emergency surgery, colostomy/ileostomy and the higher NNIS risk index score (2 or 3). The protection factors of SSI were hair removal at the night before surgery, MBP alone, laparoscopic or robotic surgery and adhesive incise drapes. Furthermore, the multivariate analysis revealed that the independent risk factors of SSI following colorectal surgery were NNIS risk index score for 2 or 3 and hypertension, while incisional protection with adhesive incise drapes and laparoscopic or robotic surgery were protective factors for SSI. In three variables of NNIS risk index score, Supplementary file 2 revealed that the surgical wound class (contaminated or dirty) was were the main predictor of SSI.

**Table 4:** Univariate and multivariate logistic regression analysis of risk factors of SSI following colorectal surgery

Variables	univariate		multivariate	
	***p*** value	OR (95% CI)	***p*** value	OR (95% CI)
Hypertension				
Yes	**0.007**	1.975(1.207–3.232)	**0.024**	1.903(1.088–3.327)
No^a^				
Tuberculosis				
Yes	**0.017**	8.972(1.475–54.559)	0.083	7.880(0.762–81.492)
No^a^				
Albumin				
≥ 3.5 g/dl^a^				
< 3.5 g/dl	**< 0.001**	2.960(1.821–4.810)	0.069	1.715(0.958–3.068)
Blood glucose				
80–150 mg/dl^a^				
> 150 mg/dl	**0.008**	2.417(1.256–4.651)	0.879	1.065(0.475–2.388)
< 80 mg/dl	0.637	1.166(0.617–2.200)		
Type of surgery				
Rectal surgery^a^				
Colon surgery	**0.002**	2.419(1.370–4.269)	0.154	0.631(0.335–1.188)
Urgency of surgery				
Elective^a^				
Emergency	**< 0.001**	5.927(3.245–10.827)	0.913	1.056(0.397–2.815)
Timing of hair removal				
None^a^				
Night before surgery	**0.009**	0.477(0.272–0.834)	0.342	0.711(0.352–1.442)
Day of surgery	0.311	0.698(0.348–1.399)		
Bowel preparation				
None^a^				
MBP only	**< 0.001**	0.376(0.223–0.634**)**	0.185	0.650(0.344–1.229)
OABP only	0.554	1.324(0.523–3.355)		
MBP + OABP	0.083	0.427(0.163–1.119)		
Approach				
Open^a^				
Laparoscopic or robotic	**< 0.001**	0.259(0.157–0.429)	**< 0.001**	0.363(0.200–0.659)
Incisional protection				
None^a^				
Wound edge protector	0.545	0.833(0.460–1.507)		
Gauze	0.368	0.676(0.288–1.585)		
Adhesive incise drapes	**0.031**	0.472(0.239–0.932)	**0.018**	0.400(0.187–0.855)
Skin closure materials				
Silk sutures^a^				
Absorbable sutures	0.060	0.524(0.268–1.027)		
Skin staples	0.953	0.978(0.466–2.051)		
other	0.053	2.496(0.988–6.307)		
Incisional wound irrigation				
None^a^				
Incisional wound irrigation	0.111	0.465(0.181–1.193)		
Hydrogen peroxide solution	0.317	0.500(0.129–1.942)		
Saline	0.134	0.562(0.264–1.195)		
Other	0.257	1.920(0.621–5.936)		
Colostomy/ileostomy				
No^a^				
Yes	**< 0.001**	3.805(2.233–6.483)	0.081	1.820(0.929–3.564)
NNIS risk index				
0 or 1^a^				
2 or 3	**< 0.001**	6.481(3.655–11.493)	**< 0.001**	3.840(1.926–7.658)

^a^reference

## Discussion

SSI is a common complication after colorectal surgery. Indeed, previous studies have reported that the incidence rate of SSI following colorectal surgery could be up to 20% [9–11]. Based on Japan nosocomial infection surveillance system national database from 2008 through 2010, the cumulative incidence rates of SSI for rectal and colon surgery were 15.0 and 17.8%, respectively [12]. Additionally, the results of American College of Surgeons National Surgical Quality Improvement Program found a reduction in colorectal SSI incidence from 17.58% of 2011 to 5.11% of 2015 [13]. This comprehensive observational study investigated the SSI incidence and risk factors of colorectal surgery among 1046 patients from 55 hospitals in China. This study demonstrated that the incidence rate of SSI following colorectal surgery was 7.1%. In addition, the occurrence of SSI remarkably increased the treatment expenses and mortality. The independent risk factor for SSI included hypertension and high NNIS risk index score, whereas using adhesive incise drapes to protect incisions and undergoing laparoscopic surgery or robotic surgery could effectively reduce the occurrence of SSI.

The present study identified that prior diagnosis of hypertension acted as an independent risk factor of SSI following colorectal surgery. Several studies have found hypertension is an important risk factor in spine operations [11], cesarean deliveries [14] and breast cancer surgery [15]. However, there is no specific study to discuss the association between SSI and hypertension after colorectal surgery. Intriguingly, it was reported that lowest postoperative mean arterial pressure was related to surgical site infection following colorectal surgery, but postoperative time-weighted mean arterial pressure was not [16]. Although hypertension was found to be the major risk factor, some details including the variation of blood pressure during the perioperative period were not recorded in our study, which could be a focus in our future research.

We also showed that NNIS risk index score for 2 or 3 was an independent predictor of SSI, which is consistent with our previous study [17]. The NNIS risk index contains the wound class, the ASA score, and the duration of surgery. The present study demonstrated that the surgical wound class (contaminated or dirty) was the main predictor of SSI among three variables of NNIS risk index score. The odds ratio of the ASA score (level of 3 or 4) was second to that of the surgical wound class (contaminated or dirty). The ASA score (level of 4 and 5) has been reported to be an important risk factor for SSI [18]. Therefore, patients with higher ASA score may be inclined to suffer more risks of SSI after surgery.

As is well-known, laparoscopic surgery and robotic surgery are becoming cumulatively common worldwide. Several studies have found that laparoscopic approach remarkably reduces SSI following colorectal surgery [19, 20]. Our study enrolled 50 patients receiving robotic surgery, two of which had SSI following surgery. The incidence rate of SSI in patients undergoing robotic surgery was 4.0%, which was similar to that undergoing laparoscopic surgery (3.6%) and was much lower than open surgery (12.8%). Thus, we combined patients accepting either robotic surgery or laparoscopic surgery into the same group, since both of them could decrease the SSI incidence by contrast with open surgery.

To better strengthen the aspects associated with wound edge isolation, devices of incisional protection have been manufactured and marketed. In this study, we found that there were three main kinds of incisional protection devices, comprising wound edge protectors, adhesive incise drapes and gauze, used for colorectal surgery in China. Although both single- and double-ring wound edge protectors have been observed to be the beneficial effect to reduce the SSI rate in previous studies [21–23], only adhesive incise drapes could be the independent protective factors for SSI in this study. Adhesive incise drapes are utilized to separate the adjacent skin surfaces from the surgical wounds, preventing the migration of microorganisms [24]. In this study, there is one deficiency whether adhesive incise drapes are impregnated with antimicrobial materials. Moreover, two randomized controlled trials demonstrated that using incise drapes without antimicrobial properties had neither benefit nor harm for the prevention of SSI [25, 26]. Intriguingly, antimicrobial-impregnated adhesive incise drapes are still controversial in reducing the risk of SSI [27, 28]. According to global guidelines for the prevention of SSI from World Health Organization (WHO) [29], it is unnecessary to use adhesive incise drapes with or without antimicrobial properties for preventing SSI, but this is a conditional recommendation with low to very low quality of evidence. Taken together, we perhaps need more high quality randomized controlled trials to evaluate the influence of adhesive incise drapes on SSI.

Preoperative bowel preparation remains a controversial and intriguing issue around the world, with much debate regarding the use of MBP combined with or without OABP. Many studies demonstrated that MBP for colorectal surgery may not prevent SSI and improve outcome for patients [30, 31]. In our study, results from univariate analysis showed the incidence of SSI in patients with MBP was significantly lower than those with no bowel preparation. However, the multivariate analysis revealed that the bowel preparation was not the independent risk factor of SSI in this study. Our previous study found that MBP combined with OABP significantly reduced SSI and minimized the readmission rates in contaminated and dirty types of colorectal surgery [32]. The global guideline from WHO suggests preoperative MBP combined with OABP should be used to reduce the risk of SSI in the patients receiving elective colorectal surgery [29]. Therefore, whether undergoing the bowel preparation and how to conduct the bowel preparation may depend on the different surgical wound class and surgical site. Furthermore, we found that 29 out of 331 patients (8.8%) with no bowel preparation had contaminated or dirty wounds, and 13 out of 575 patients (2.3%) receiving MBP had contaminated or dirty wounds in this study, which could account for the reason that patients receiving no bowel preparation had the higher SSI incidence. In addition, the most common method of bowel preparation was MBP alone (SSI rates: 4.5%), only 42 patients received OABP (SSI rates: 14.3%) and 98 patients underwent MBP with OABP (SSI rates: 5.1%) in this study. Limited by the small number of cases (OABP with or without MBP), our study did not clearly reflect the difference among the bowel preparation of OABP with or without MBP. More cases should be collected to analyze the role of different bowel preparation in SSI after colorectal surgery. However, our study indicated that it was not very common to using oral antibiotics for the bowel preparation of colorectal surgery in China.

There are many studies reporting that rectal operations had a higher risk than colectomies [33, 34]. Although rectal operations had a lower risk compared with colonic operations in this study, the multivariate analysis revealed that type of surgery was not the independent risk factor (*P* = 0.182) in Table 4. Our data included the results of 2 years of cross-sectional studies (2018 and 2019). In 2018, the SSI incidence rate for colon surgery was 8.2% and the SSI incidence rate for rectal surgery was 3.8%. In 2019, the SSI incidence rate for colon surgery and rectal surgery was 9.0 and 4.2%, respectively. The results from 2 years of cross-sectional studies remain similar. Furthermore, 44 out of 641 patients (6.9%) undergoing colon surgery had contaminated or dirty wounds, and 10 out of 405 patients (2.5%) receiving rectal surgery had contaminated or dirty wounds in this study, which could lead to increased SSI incidence in colon surgery.

In this study, we carried out follow-up via standard telephone interview or review of the readmission records if patients were discharged from hospital after less than postoperative 30 d. Data of patients who were lost to follow-up would not be included. Although there was withdraw bias, most patients have been followed up in this study. Therefore, our results are reliable. In addition, some limitations existed in the present study. Firstly, we only included patients receiving colorectal surgery from May 1 to June 30 of 2018 and the same time of 2019. Some bias may be generated due to data collected during such a short period. Secondly, some details need to be improved, including the perioperative variation of blood pressure and different types of wound edge protectors and adhesive incise drapes.

## Conclusions

Our study demonstrated that the nationwide incidence rate of SSI after colorectal surgery is 7.1%. The prior diagnosis of hypertension, incisional protection (adhesive incise drapes), laparoscopic or robotic surgery, and NNIS risk index score of 2 or 3 may be associated with SSI occurrence after colorectal surgery. Considering drawbacks of this observational study, some other studies, such as randomized controlled trials, are needed to further identify risk factors for SSI following colorectal surgery. To further know nationwide SSI occurrence after colorectal surgery, the multicenter study to found more predictors for SSI in the following years should be conducted.

## Supplementary Information

**Additional file 1.**

**Additional file 2.**

## Data Availability

The datasets generated in the current study are not publicly available. However, the datasets used or analyzed in the current study will be available from the corresponding author on reasonable request.

## Abbreviations

SSI: Surgical site infection
BMI: Body mass index
ASA score: American Society of Anesthesiologists physical status score
OABP: Oral antibiotics bowel preparation
MBP: Mechanical bowel preparation
The NNIS risk index: The national nosocomial infections surveillance risk index
SD: Standard deviation
IQR: Interquartile range
