# Supplementary material for: Incidence and risk factors of surgical site infection following colorectal surgery in China: a national cross-sectional study
Source: BMC Infect Dis. 2020 Nov 12;20:837. doi: 10.1186/s12879-020-05567-6 (PMC7663877; doi:10.1186/s12879-020-05567-6)
Supplement: Supplementary file 1 — Additional file 1. [file 12879_2020_5567_MOESM1_ESM.doc]

**Supplementary file 1**

| **Included hospital** |
| --- |
| Jinling hospital |
| The affiliated hospital of qingdao university |
| Guangdong provincial people's hospital |
| West china hospital |
| Hunan provincial people's hospital |
| People's hospital of guangxi zhuang autonomous region |
| The first affiliated hospital of nanchang university |
| People's hospital of Xinjiang uygur autonomous region |
| Shangdong provincial hospital |
| The second Xiangya hospital of central south university |
| Nanyang center hospital |
| The affiliated hospital of yangzhou university |
| Chinese PLA general hospital |
| Shangqiu first people's hospital |
| Zhangjiagang first people's hospital |
| 900 Hospital of the PLA |
| Chenzhou first people's hospital |
| Wuhan general hospital of the PLA |
| Yantai Yuhuangding hospital |
| Zigong first people's hospital |
| The first affiliated hospital of Zhengzhou university |
| Yueyang first people's hospital |
| Huangshan shoukang hospita |
| Taizhou first people's hospital |
| Beijing tsinghua changgung hospital |
| The affiliated hospital of Xinjiang medical university |
| The affiliated Zhongshan hospital of xiamen university |
| The affiliated hospital of Xuzhou medical university |
| Baotou central hospital |
| Dongguan kanghua hospital |
| Tianjin medical university general hospital |
| Shengli oilfield central hospital |
| Dongying people's hospital |
| Tianjin first central hospital |
| The second hospital of jilin university |
| The second affiliated hospital of university of south China |
| general hospital of huainan eastern hospital group |
| Hunan provincial tumor hospital |
| 901 Hospital of the PLA |
| The affiliated Sir Run Run Shaw hospital of Zhejiang University |
| The second hospital of Lanzhou university |
| The second affiliated hospital of PLA army medical university |
| Xishan people's hospital |
| Lanzhou general hospital of the PLA |
| The fourth affiliated hospital of Jilin university |
| The first affiliated hospital of Changzhi medical college |
| Liyang people's hospital |
| Zigong fourth people's hospital |
| Xuzhou first people's hospital |
| Shenzhen people's hospital |
| Shaoxing central hospital |
| The first hospital of Shanxi medical university |
| Nanchong central hospital |
| Hefei second people's hospital |
| The second affiliated hospital of Dalian medical university |
