# Supplementary material for: Incidence and risk factors of surgical site infection following colorectal surgery in China: a national cross-sectional study
Source: BMC Infect Dis. 2020 Nov 12;20:837. doi: 10.1186/s12879-020-05567-6 (PMC7663877; doi:10.1186/s12879-020-05567-6)
Supplement: Supplementary file 2 — Additional file 2. [file 12879_2020_5567_MOESM2_ESM.doc]

**Supplementary file 2**

| **Variables** | **univariate** | | **multivariate** | |
| --- | --- | --- | --- | --- |
|  | **p value** | **OR (95% CI)** | **p value** | **OR (95% CI)** |
| ASA score |  |  |  |  |
| 1 or 2* |  |  |  |  |
| 3 or 4 | **0.001** | 2.350(1.432-3.854) | **0.006** | 2.035(1.226-3.380) |
| Surgical wound class |  |  |  |  |
| Clean-contaminated* |  |  |  |  |
| Contaminated or dirty | **<0.001** | 5.437(2.804-10.542) | **<0.001** | 4.780(2.437-9.378) |
| Surgical duration | **0.039** | 1.002(1.000-1.004) | **0.023** | 1.002(1.000-1.004) |

*: reference
